# Supplementary material for: From sequence to enzyme mechanism using multi-label machine learning
Source: BMC Bioinformatics. 2014 May 19;15:150. doi: 10.1186/1471-2105-15-150 (PMC4229970; doi:10.1186/1471-2105-15-150)
Supplement: Additional file 2 — Java code of ml2db. Additional file ml2db_code.tar.gz contains the Java source code to run the multi-label machine learning experiments and save the results to database. The code’s Javadoc is included. [file 1471-2105-15-150-S2.zip › additional file 2/ml2db/ml2db/doc/mulan/data/class-use/LabelSet.html]

Uses of Class mulan.data.LabelSet


JavaScript is disabled on your browser.


- Overview
- Package
- Class
- Use
- Tree
- Deprecated
- Index
- Help

- Prev
- Next

- Frames
- No Frames

- All Classes

## Uses of Class mulan.data.LabelSet

- Packages that use LabelSet

  | Package | Description |
  | --- | --- |
  |  |  |
  | --- | --- |
  | mulan.data |  |
- - ### Uses of LabelSet in mulan.data

    Methods in mulan.data that return LabelSet

    | Modifier and Type | Method and Description |
    | --- | --- |
    |  |  |
    | --- | --- |
    | `static LabelSet` | LabelSet.`fromBitString(java.lang.String bits)` Constructs a LabelSet object from a bitstring. |
    | `static LabelSet` | LabelSet.`intersection(LabelSet l1, LabelSet l2)` |

    Methods in mulan.data that return types with arguments of type LabelSet

    | Modifier and Type | Method and Description |
    | --- | --- |
    |  |  |
    | --- | --- |
    | `java.util.ArrayList<LabelSet>` | LabelSet.`getSubsets()` Constructs all subsets of a labelset (apart from the empty one). |
    | `java.util.HashMap<LabelSet,java.lang.Integer>` | Statistics.`labelCombCount()` returns the HashMap containing the distinct labelsets and their frequencies |
    | `java.util.Set<LabelSet>` | Statistics.`labelSets()` returns a set with the distinct labelsets of the dataset |

    Methods in mulan.data with parameters of type LabelSet

    | Modifier and Type | Method and Description |
    | --- | --- |
    |  |  |
    | --- | --- |
    | `int` | LabelSet.`compareTo(LabelSet o)` Used for sorting collections of labelsets according to size |
    | `int` | LabelSet.`hammingDifference(LabelSet other)` Calculates the Hamming Distance between the current labelset and another labelset. |
    | `static LabelSet` | LabelSet.`intersection(LabelSet l1, LabelSet l2)` |
    | `int` | Statistics.`labelFrequency(LabelSet x)` returns the frequency of a labelset in the dataset |

- Overview
- Package
- Class
- Use
- Tree
- Deprecated
- Index
- Help

- Prev
- Next

- Frames
- No Frames

- All Classes
